# Serum Gamma-Glutamyl Transferase and Ferritin Synergistically Associated with the Rate of Chronic Kidney Disease

**DOI:** 10.1155/2017/9765259

**Published:** 2017-06-04

**Authors:** Tao Chen, Yan Ren, Yun Gao, Haoming Tian

**Affiliations:** Department of Endocrinology and Metabolism, West China Hospital of Sichuan University, Chengdu 610041, China

## Abstract

The present study investigated the effects of GGT and SF on the risk of CKD. 1024 participants (436 men and 588 women) were divided into three groups according to GGT and SF levels: group 1 (both GGT and SF not in the fourth quartile), group 2 (only GGT or SF in the fourth quartile), and group 3 (both GGT and SF in the fourth quartile). The risks of CKD in different levels of GGT and SF and in groups 2-3 compared with group 1 were analyzed by multiple logistic regression. GGT or SF in the highest quartile was associated with increased risk of CKD. Such associations attenuated after adjustment for confounding factors. The incidences of CKD, especially albuminuria, increased across the three groups. Correspondingly, malondialdehyde (MDA) levels gradually increased from group 1 to group 3. The risks of CKD were higher in groups 2 and 3 than that in group 1. In group 3, the increased rate was independent of age, BMI, alcohol drinking, diabetes mellitus, hypertension, hypertriglyceridemia, and metabolic syndrome (odds ratios from 1.887 to 2.293, *P* < 0.05). In summary, this study suggested that GGT and SF synergistically influence the rate of CKD.

## 1. Introduction

The incidence of chronic kidney disease (CKD) is increasing in recent decades in China and in other parts of the world [1, 2]. CKD is one of the leading causes for kidney failure and contributes to systemic pathophysiological processes and, thus, is becoming a great burden to public health [2, 3].

Unfortunately, the etiology of CKD is still undetermined. Risk factors of CKD included hypertension, hyperglycemia, hyperuricemia, and metabolic syndrome [1, 4, 5]. However, the exact link between CKD and these factors is unclear. A speculation is that both CKD and the aforementioned risk factors were caused by the same underlying mechanism [2, 6].

Oxidative stress is recognized as common soil of many noninfectious chronic diseases including CKD [7, 8]. Serum gamma-glutamyl transferase (GGT) and serum ferritin (SF) are two frequently used biomarkers in clinical practice. GGT cleavages glutathione, one of the most important extracellular antioxidative agents. This process will lead to increase production of reactive oxygen species [9]. SF is an index of body iron storage. Iron in the form of Fe(III) and Fe(II) is necessary for GGT's action on the catabolism glutathione [9]. Therefore, GGT and SF could have synergetic effect on oxidative stress and further affect the risk of CKD. Our previous study showed that serum GGT was correlated with SF, and they could synergistically affect the risk of type 2 diabetes [10]. Several but not all studies showed that GGT was associated with increased risk of CKD [11–13]. Few studies analyzed the association between SF and CKD, especially the interaction between GGT and SF on CKD. To clarify these issues, the present study employed the data from a recent survey in a Chinese minority and further investigated the potential effects of GGT and SF.

## 2. Methods

### 2.1. Study Design

This was a cross-sectional study initially designed to investigate the prevalence of type 2 diabetes in Sichuan province, including Xichang area where Yi nationality population inhabits, an old minor nationality in West South of China.

### 2.2. Participants

All participants included in this study were from Yi nationality. Details of this population and study design were described in a previously published article [10]. In brief, a total of 1288 individuals of Yi nationality, 20–74 years of old, participated in a nationwide survey. 1024 subjects (84.0%) with integral data were enrolled. Patients were excluded if they were previously diagnosed with type 2 diabetes (T2D), type 1 diabetes, or other special type diabetes, previously diagnosed liver-biliary disease. This study was a part of a national survey of the prevalence of type 2 diabetes and metabolic syndrome in China, which was approved by China-Japan Friendship Hospital's Drugs/Medical Apparatus & Instruments Ethics Committee (07020470055), and all subjects gave their informed consent. This study was registered on the website of Chinese clinical trial registry (TR-CCH-ChiCTR-CCH-00000361).

### 2.3. Measurements

At the site of survey, a fasting morning spot urine sample was collected, stored at −20°C for less than 2 months, and then was transported in package conditioned by dry ice to China-Japan Friendship Hospital in Beijing for measurement. Serum GGT, albuminuria, and urinary creatinine were measured according to standard laboratory procedures (with immunoturbidimetric tests and Jaffe's kinetic method, resp.). The urinary albumin to creatinine ratio (ACR; mg/g creatinine) was calculated. SF level was measured with a radioimmunoassay kit (Beijing North Institute of Biological Technology). Serum creatinine level was measured at the survey site by Hitachi automatic biochemical analyzer. Estimated glomerular filtration rate (eGFR) was calculated according to a formula described in previous studies [1, 5]. Malondialdehyde (MDA) as a marker of oxidative stress was measured by MDA detection kit (Nanjing Jiancheng Bioengineering Institute).

### 2.4. Definition

Patients with an ACR more than 30 mg/g were defined as having albuminuria (no patients in this study had an ACR more than 300 mg/g). Chronic kidney disease is defined by microalbuminuria or eGFR less than 60 mL/min per 1.73 m^2^ as described in the previous studies [1, 5].

## 3. Statistical Analysis

Serum levels of GGT and SF were firstly divided into quartiles with values specific to each gender from each geological location (urban or rural). Then, all subjects were reconstituted into three groups: group 1 (both GGT and SF were not in the fourth quartile), group 2 (only GGT or SF was in the fourth quartile), and group 3 (both GGT and SF were in the fourth quartile). The odds ratios (OR) and 95% confidential interval (95% CI) of CKD in different levels of GGT and SF, and in groups 2 and 3 compared with group 1, were assessed by multiple logistic regressions. The differences in other categorical variables were analyzed with chi-square tests, and those in continuous variables were compared by Student's *t*-tests or one-way ANOVA. All *P* values were two tailed, and those *α* < 0.05 were considered statistically significant.

## 4. Results

### 4.1. Demographic and Clinical Characteristics of the Participants in the Study

Overall, participants from low to high quartiles of GGT or SF tended to be older, obese, with high levels of triglyceride, MDA, urinary albumin, and high incidences of diabetes mellitus, hypertension, and metabolic syndrome (Tables 1 and 2).

As shown in Table 3, participants were older and with higher BMI from group 1 to group 3. There were more drinkers and heavy drinkers in group 3 than in group 1 and group 2. The prevalence of diabetes mellitus, hypertension, and metabolic syndrome and the levels of triglyceride and MDA gradually increased from group 1 to group 3.

### 4.2. The Relationship between GGT and SF

As shown in our previous study, GGT was positively associated with SF (*r* = 0.237 to 0.303, all *P* < 0.05), which was independent of BMI [10].

### 4.3. CKD Incidences in Different Levels of GGT or SF

CKD rates were higher in the third and fourth quartiles of GGT when compared with the lowest quartile (OR 1.422, 95% CI 1.106, 1.830; OR 1.364, 95% CI 1.156, 1.610). Such associations were attenuated when adjusted for age and BMI plus diabetes mellitus, hypertension, or metabolic syndrome (Table 4).

CKD rates were higher in the fourth quartile of SF when compared with the lowest quartile (OR 1.373, 95% CI 1.174, 1.606). Such an association was attenuated but still existed when adjusted the above confounding factors (Table 5).

### 4.4. CKD Incidence and MDA Levels in Different Groups of GGT and SF Combination

**Figure 1:**
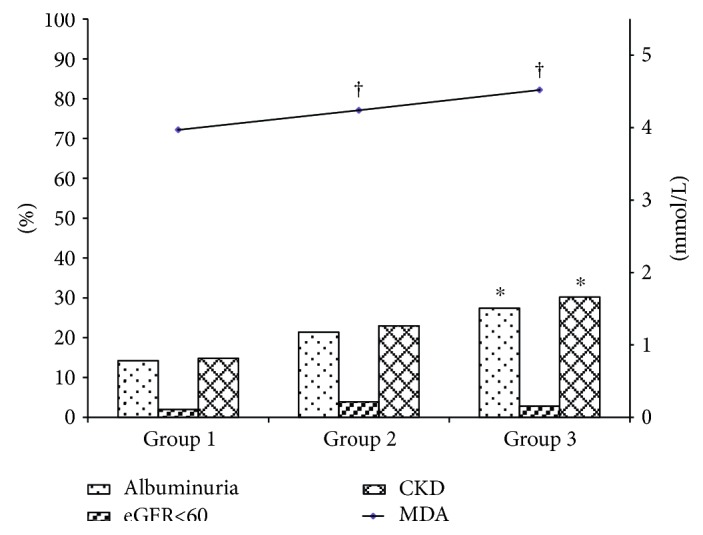
The prevalence of CKD in the different groups according to different combination of GGT and SF quartiles. Group 1: both GGT and SF were not in the fourth quartile; group 2: only GGT or SF was in the fourth quartile; group 3: both GGT and SF were in the fourth quartile. ^†^*P* < 0.05 compared with group 1; ^∗^*P* < 0.05 compared across groups.

ACR levels were significantly higher in groups 2 and 3 than in group 1 and was higher in group 3 than in group 2 but with no significant difference. Similarly, eGFR levels were significantly lower in group 2 and group 3 compared with group 1, and no significant difference was observed between group 2 and group 3 (Table 3). Moreover, the rates of microalbuminuria (14.2%, 21.4%, and 27.4% in groups 1 to 3, resp., *P* < 0.01) and CKD (14.8%, 23.0%, and 30.2% in groups 1 to 3, resp., *P* < 0.01) were significantly increased from group 1 to group 3, while that of reduced eGFR (less than 60 mL/min per 1.73 m^2^) was not different across groups (Figure 1).

### 4.5. Synergetic Effects of GGT and SF on the Rates of CKD

The results of logistic regression showed that CKD rates increased in groups 2 and 3 compared with group 1 (OR 1.743; 95% CI 1.228, 2.474; and OR 2.413; 95% CI 1.504, 3.871). The ORs were independent of age and BMI but attenuated after adjustment for heavy drinking, diabetes mellitus, hypertension, hypertriglyceridemia, and metabolic syndrome, respectively, in group 2, and slightly attenuated but still be significant in group 3 (OR from 1.887 to 2.293, *P* < 0.05; Table 6). This synergy appears to be a novel underlining mechanism for CKD.

## 5. Discussion

The present study found that subjects with high level of GGT or SF had increase prevalence of CKD compared with those with relatively low levels of GGT and SF. When GGT and SF were both at high levels, the CKD rates were the highest which were independent of age, BMI, drinking status, diabetes mellitus, hypertension, hypertriglyceridemia, and metabolic syndrome. The increased rates of CKD from group 1 to group 3 were accompanied by gradual increased levels of MDA.

Several published studies had investigated the association between GGT and CKD. Some studies showed that serum GGT within the physiologic range independently predicted albuminuria among patients with or without hypertension or diabetes [11, 12], while another study showed that there was no association between increased levels of serum GGT and CKD [13]. The discrepancy among these studies was unclear. What is clear is that most of these studies were based on the same speculation that the effect of GGT is related to oxidative stress. GGT plays important roles in GSH metabolism, and the latter is the most important extracellular antioxidative agent. In the process of GGT-mediated cleavage of glutathione, iron exerts important effect as an electron transporter [9]. Theoretically, the circulatory iron could interact with GGT, lead to enhanced oxidative stress, and increase the risk of CKD. Unfortunately, few study investigated the association between SF and CKD. In the present study, SF in the highest quartile was associated with increased risk of CKD, but such an association was dependent on confounding factors such as drinking status, diabetes mellitus, hypertension, hypertriglyceridemia, or metabolic syndrome. However, when GGT and SF were analyzed in combination, it can be seen that the incidence of CKD was the highest in group 3 where GGT and SF were both at the highest quartile and which was accompanied by the highest MDA level. These data highly suggested that GGT and SF worked together to promote increased oxidative stress and independently led to higher rate of CKD.

A subgroup analysis from the Nurses' Health Study showed that western diet (featured by higher portion of red and processed meats, saturated fats, and sweets) is associated with a significantly increased odds of microalbuminuria and rapid kidney function decrease [14]. High intake of red meat was also associated with elevated GGT and SF levels [15, 16]. So, it could be proposed that excessive red meat intake could lead to increased serum concentration of GGT and SF and thus increased the risk of CKD.

The major limitation of this study is its cross-sectional design, which could only suggest potential association between GGT, SF, oxidative stress, and CKD. Another limitation is that the sample size of the population was not large enough when both GGT and SF were taken into account, which hampers us to further analyze according to different gender and location; for example, GGT and SF synergy might be more obvious in men (Supplementary Tables 1 and 2 available online at https://doi.org/10.1155/2017/9765259). Third, due to insufficient blood sample, only MDA was measured as a marker of oxidative stress. This prevented us from analyzing the reason that the CKD risks in groups 2 and 3 were not dramatically weakened after adjustment for MDA level. Fourth, a large portion of participants were alcohol drinkers. Alcohol drink could increase serum levels of GGT and SF. Although the association between GGT, SF, and CKD still existed after adjustment for alcohol drinking or heavy drinking, potential interference might still exist.

## 6. Conclusions

In conclusion, this study found that GGT and SF could act together to increase the rate of CKD. The potential mechanism might be related to enhanced oxidative stress. Prospective studies or delicate designed animal studies are warranted to further investigate the potential values of GGT and SF in clinical practice with CKD.

## Supplementary Material

Table S1 The risks of CKD in groups with the highest quartile of serum GGT or/and ferritin compared with group with other quartiles of serum GGT and ferritin levels in men. Table S2 The risks of CKD in groups with the highest quartile of serum GGT or/and ferritin compared with group with other quartiles of serum GGT and ferritin levels in women.

## Figures and Tables

**Table 1:** General characteristics of individuals stratified by different levels of GGT.

	Men				Women			
	Quartile 1	Quartile 2	Quartile 3	Quartile 4	Quartile 1	Quartile 2	Quartile 3	Quartile 4
*n*	94	122	109	111	133	144	162	149
Age, years (S. E.)	43.2 (1.9)	44.6 (1.4)	48.6 (1.3)^†^	43.7 (1.1)	42.6 (1.4)	42.6 (1.1)	45.8 (1.1)^†‡^	45.7 (1.0)^‡^
BMI, kg/m^2^	20.7 (0.3)	21.4 (0.3)	22.4 (0.3)^†‡^	24.0 (0.3)^†‡§^	20.6 (0.2)	21.7 (0.3)^†^	22.4 (0.3)^†‡^	24.5 (0.3)^†‡§^
Drinking, % (*n*)	46.8 (44)	59.0 (72)	61.5 (67)	71.2 (79)^∗^	12.0 (16)	15.3 (22)	13.6 (22)	21.5 (32)
Heavy drinking^b^, % (*n*)	40.4 (38)	47.5 (58)	50.5 (55)	64.0 (71)^∗^	8.3 (11)	6.3 (9)	3.7 (6)	13.4 (20)^∗^
Diabetes mellitus^c^, % (*n*)	7.4 (7)	6.6 (8)	8.3 (9)	12.6 (14)	1.5 (2)	4.2 (6)	4.3 (7)	10.1 (15)^∗^
Hypertension^d^, % (*n*)	7.4 (7)	18.0 (22)	28.4 (31)	28.8 (32)^∗^	11.3 (15)	11.8 (17)	19.1 (31)	25.5 (38)^∗^
TG, mmol/L	1.3 (0.1)	1.2 (0.1)	1.6 (0.1)	2.6 (0.3)^†‡§^	1.3 (0.1)	1.3 (0.1)	1.5 (0.1)	2.2 (0.1)^†‡§^
Metabolic syndrome^e^, % (*n*)	4.3 (4)	5.7 (7)	14.7 (16)	23.4 (26)^∗^	8.3 (11)	11.8 (17)	21.0 (34)	42.3 (63)^∗^
SF, *μ*mol/L	130.2 (70.9, 166.3)	158.0^†^ (124.3, 253.8)	193.5^†‡^ (127.6, 288.4)	270.6^†‡§^ (180.6, 332.1)	43.4 (27.9, 96.8)	49.7 (23.7, 101.6)	81.8^†‡^ (35.6, 156.3)	104.3^†‡§^ (56.2, 157.4)
GGT, U/L	13 (10, 14)	21^†^ (17, 29)	36^†‡^ (29, 48)	105.5^†‡§^ (65.8, 172.0)	9 (8, 11)	14^†^ (12, 18)	21^†‡^ (17, 27.5)	50^†‡§^ (38, 82)
MDA, mmol/L	4.4 (0.2)	4.3 (0.1)	4.4 (0.1)	4.5 (0.1)	3.7 (0.1)	3.7 (0.1)	4.0 (0.1)^†‡^	4.1 (0.1)^†‡^
eGFR, mL/min per 1.73 m^2^	96.7 (1.8)	91.4 (2.1)	91.5 (2.6)	89.6 (1.5)^†^	90.0 (1.2)	90.4 (1.3)	86.0 (1.3)^†‡^	86.2 (1.3)^†‡^
ACR, mg/g creatinine	10.1 (4.9, 19.7)	10.1 (3.3, 19.8)	11.0 (4.5, 20.7)	9.3 (4.2, 29.3)	10.1 (5.3, 15.5)	10.4 (3.6, 22.5)	11.2 (5.7, 23.4)	14.4^†‡^ (5.8, 28.3)

^†^ *P* < 0.05 compared with quartile 1; ^‡^*P* < 0.05 compared with quartile 2; ^§^*P* < 0.05 compared with quartile 3.^∗^*P* < 0.05 compared across all quartiles. ^b^Drinking more than 60 mL of alcohol per day. ^c^Diabetes mellitus was defined according to the criteria of the American Diabetes Association (1999). ^d^Hypertension was defined as systolic blood pressure ≥ 140 mmHg and/or diastolic blood pressure ≥ 90 mmHg. ^e^Metabolic syndrome was defined according to the criteria of the IDF (2005).

**Table 2:** General characteristics of individuals stratified by different levels of SF.

	Men				Women			
	Quartile 1	Quartile 2	Quartile 3	Quartile 4	Quartile 1	Quartile 2	Quartile 3	Quartile 4
*n*	108	109	110	109	146	147	148	147
Age, years (S. E.)	42.9 (1.6)	45.0 (1.5)	44.6 (1.3)	47.8 (1.2)^†^	35.1 (0.8)	40.0 (1.0)^†^	45.8 (1.0)^†‡^	56.4 (0.9)^†‡§^
BMI, kg/m^2^	20.9 (0.3)	21.7 (0.3)	22.4 (0.3)^†^	23.6 (0.3)^†‡§^	22.2 (0.3)	21.6 (0.3)	23.0 (0.3)^‡^	22.7 (0.4)^‡^
Drinking, % (*n*)	51.9 (56)	62.4 (68)	53.6 (59)	72.5 (79)^∗^	15.1 (22)	16.3 (24)	15.5 (23)	15.6 (23)
Heavy drinking^b^, % (*n*)	40.7 (44)	55.0 (60)	46.4 (51)	61.5 (67)^∗^	6.2 (9)	7.5 (11)	8.8 (13)	8.8 (13)
Diabetes mellitus^c^, % (*n*)	7.4 (8)	11.0 (12)	6.4 (7)	10.1 (11)	2.7 (4)	2.7 (4)	5.4 (8)	9.5 (14)^∗^
Hypertension^d^, % (*n*)	13.9 (15)	22.9 (25)	19.1 (21)	28.4 (31)	11.6 (17)	8.2 (12)	20.3 (30)	28.6 (42)^∗^
TG, mmol/L	1.3 (0.1)	1.4 (0.1)	1.6 (0.2)	2.5 (0.3)^†‡§^	1.2 (0.1)	1.4 (0.1)^†^	1.6 (0.1)^†^	2.1 (0.1)^†‡§^
Metabolic syndrome^e^, % (*n*)	4.6 (5)	11.0 (12)	11.8 (13)	21.1 (23)^∗^	13.0 (19)	10.9 (16)	23.6 (35)	37.4 (55)^∗^
SF, *μ*mol/L	71.7 (54.0, 95.2)	143.1^†^ (128.2, 174.1)	210.0^†‡^ (172.5, 274.8)	340.4^†‡§^ (306.1, 379.7)	18.5 (10.3, 27.3)	44.8^†^ (37.4, 56.9)	99.9^†‡^ (80.6, 117.3)	202.0^†‡§^ (152.0, 262.1)
GGT, U/L	19 (13, 31.5)	26^†^ (16, 40.5)	34^†‡^ (20, 65.8)	53.5^†‡§^ (29.2, 121.8)	14 (11, 22)	16 (11, 28.5)	19^†‡^ (12, 32)	25^†‡§^ (14, 47)
MDA, mmol/L	4.2 (0.1)	4.1 (0.1)	4.4 (0.1)	4.9 (0.1)^†‡§^	3.6 (0.1)	3.8 (0.1)	4.0 (0.1)^†^	4.1 (0.1)^†‡^
eGFR, mL/min per 1.73 m^2^	95.8 (1.8)	91.7 (2.3)	91.9 (2.7)	88.9 (1.3)^†^	94.1 (1.2)	90.5 (5.2)^†^	86.2 (1.2)^†‡^	81.4 (1.4)^†‡§^
ACR, mg/g creatinine	8.6 (3.3, 19.4)	12.2 (4.1, 20.3)	10.4 (4.4, 16.9)	11.4^†^ (5.1, 34.5)	9.8 (3.6, 15.5)	10.3 (5.0, 26.0)	11.9 (5.6, 23.0)	14.6^†^ (6.6, 28.0)

^†^ *P* < 0.05 compared with quartile; ^‡^*P* < 0.05 compared with quartile 2; ^§^*P* < 0.05 compared with quartile 3; ^∗^*P* < 0.05 compared across all quartiles. ^b^Drinking more than 60 mL of alcohol per day. ^c^Diabetes mellitus was defined according to the criteria of the American Diabetes Association (1999). ^d^Hypertension was defined as systolic blood pressure ≥ 140 mmHg and/or diastolic blood pressure ≥ 90 mmHg. ^e^Metabolic syndrome was defined according to the criteria of the IDF (2005).

**Table 3:** General characteristics of individuals in groups stratified by different combinations of GGT and SF levels^a^.

	Group 1	Group 2	Group 3
*n*	614	304	106
Age, years (S. E.)	41.9 (0.6)	48.4 (0.7)^†^	49.3 (1.2)^†^
Male, % (*n*)	56.4 (346)	38.2 (116)	49.1 (52)
BMI, kg/m^2^	21.4 (0.1)	23.3 (0.2)^†^	24.3 (0.4)^†‡^
Drinking, % (*n*)	31.3 (192)	36.5 (111)	48.1 (51)^∗^
Heavy drinking^b^, % (*n*)	23.1 (142)	26.6 (81)	42.5 (45)^∗^
Diabetes mellitus^c^, % (*n*)	4.9 (30)	7.2 (22)	15.1 (16)^∗^
Hypertension^d^, % (*n*)	14.2 (87)	22.7 (69)	34.9 (37)^∗^
TG, mmol/L	1.26 (0.0)	1.98 (0.1)^†^	2.81 (0.3)^†‡^
Metabolic syndrome^e^, % (*n*)	8.1 (50)	29.3 (89)	36.8 (39)^∗^
SF, *μ*mol/L	72.5 (35.9, 131.0)	164.6 (96.2, 269.3)^†^	300.8 (211.2, 359.2)^†‡^
GGT, U/L	16 (12, 23.3)	34 (20, 63.5)^†^	75 (49.8, 127.3)^†‡^
MDA, mmol/L	4.0 (0.1)	4.2 (0.1)^†^	4.5 (0.1)^†^
eGFR, mL/min per 1.73 m^2^	92.2 (0.8)	86.1 (0.9)^†^	86.1 (1.7)^†^
ACR, mg/g creatinine	10.3 (4.0, 19.2)	12.2 (5.8, 24.2)^†^	14.4 (5.4, 35.0)^†^

^†^ *P* < 0.05 compared with group 1; ^‡^*P* < 0.05 compared with group 2; ^∗^*P* < 0.05 compared across groups. ^a^Group 1: both GGT and SF were not in the fourth quartile; group 2: only GGT or SF was in the fourth quartile; group 3: both GGT and SF were in the fourth quartile. ^b^Drinking more than 60 mL of alcohol per day. ^c^Diabetes mellitus was defined according to the criteria of the American Diabetes Association (1999). ^d^Hypertension was defined as systolic blood pressure ≥ 140 mmHg and/or diastolic blood pressure ≥ 90 mmHg. ^e^Metabolic syndrome was defined according to the criteria of the IDF (2005).

**Table 4:** The risks of CKD in groups with different levels of GGT.

	Quartile 1	Quartile 2	Quartile 3	Quartile 4
Model 0	1	1.457 (0.861, 2.464)	1.422 (1.106, 1.830)	1.364 (1.156, 1.610)
Model 1	1	—	1.360 (1.003, 1.699)	1.220 (1.012, 1.471)
Model 2	1	—	1.297 (0.997, 1.689)	1.215 (1.007, 1.466)
Model 3	1	—	1.303 (1.001, 1.697)	1.163 (0.960, 1.409)
Model 4	1	—	1.293 (0.992, 1.686)	1.178 (0.973, 1.427)
Model 5	1	—	1.302 (1.001, 1.695)	1.216 (1.007, 1.467)
Model 6	1	—	1.302 (1.000, 1.695)	1.200 (0.993, 1.451)
Model 7	1	—	1.308 (1.005, 1.703)	1.217 (1.009, 1.468)

Model 0, no confounding factors were adjusted; model 1, adjusted for age and BMI; model 2, model 1 plus drinking; model 3, model 1 plus T2D; model 4, model 1 plus hypertension; model 5, model 1 plus hypertriglyceridemia; model 6, model 1 plus metabolic syndrome; model 7, model 1 plus MDA.

**Table 5:** The risks of CKD in groups with different levels of SF.

	Quartile 1	Quartile 2	Quartile 3	Quartile 4
Model 0	1	1.551 (0.945, 2.544)	1.278 (1.000, 1.174)	1.373 (1.174, 1.606)
Model 1	1	—	1.103 (0.850, 1.431)	1.259 (1.051, 1.510)
Model 2	1	—	—	1.265 (1.053, 1.519)
Model 3	1	—	—	1.246 (1.036, 1.499)
Model 4	1	—	—	1.248 (1.040, 1.498)
Model 5	1	—	—	1.250 (1.038, 1.504)
Model 6	1	—	—	1.242 (1.034, 1.492)
Model 7	1	—	—	1.253 (1.038, 1.512)

Model 0, no confounding factors were adjusted; model 1, adjusted for age and BMI; model 2, model 1 plus drinking; model 3, model 1 plus T2D; model 4, model 1 plus hypertension; model 5, model 1 plus hypertriglyceridemia; model 6, model 1 plus metabolic syndrome; model 7, model 1 plus MDA.

**Table 6:** The risks of CKD in groups with the highest quartile of serum GGT and/or SF compared with group with other quartiles of serum GGT and SF levels^a^.

	Group 1	Group 2	Group 3
Model 0	1	1.743 (1.228, 2.474)	2.413 (1.504, 3.871)
Model 1	1	1.440 (1.000, 2.075)	2.227 (1.355, 3.662)
Model 2	1	1.438 (0.998, 2.072)	2.293 (1.382, 3.805)
Model 3	1	1.429 (0.990, 2.062)	2.051 (1.233, 3.413)
Model 4	1	1.376 (0.951, 1.991)	1.996 (1.201, 3.317)
Model 5	1	1.286 (0.878, 1.882)	1.887 (1.104, 3.226)
Model 6	1	1.267 (0.867, 1.852)	2.079 (1.241, 3.482)
Model 7	1	1.461 (1.012, 2.109)	2.215 (1.342, 3.658)

^a^Group 1: both GGT and SF were not in the fourth quartile; group 2: only GGT or SF was in the fourth quartile; group 3: both GGT and SF were in the fourth quartile. Model 0, no confounding factors were adjusted; model 1, adjusted for age and BMI; model 2, model 1 plus drinking; model 3, model 1 plus T2D; model 4, model 1 plus hypertension; model 5, model 1 plus hypertriglyceridemia; model 6, model 1 plus metabolic syndrome; model 7, model 1 plus MDA.
