# Supplementary material for: Serum Gamma-Glutamyl Transferase and Ferritin Synergistically Associated with the Rate of Chronic Kidney Disease
Source: Dis Markers. 2017 Jun 4;2017:9765259. doi: 10.1155/2017/9765259 (PMC5474265; doi:10.1155/2017/9765259)
Supplement: Supplementary file 1 — Table S1 The risks of CKD in groups with the highest quartile of serum GGT or/and ferritin compared with group with other quartiles of serum GGT and ferritin levels in men. Table S2 The risks of CKD in groups with the highest quartile of serum GGT or/and ferritin compared with group with other quartiles of serum GGT and ferritin levels in women. [file 9765259.f1.docx]

Table S1 The risks of CKD in groups with the highest quartile of serum GGT or/and ferritin compared with group with other quartiles of serum GGT and ferritin levels in men^a^

|  | Group 1 | Group 2 | Group 3 |
| --- | --- | --- | --- |
| Model 0 | 1 | 2.095(1.181, 3.716) | 2.753(1.360,5.570) |
| Model 1 | 1 | 2.070(1.166,3.676) | 2.759(1.363,5.585) |
| Model 2 | 1 | 1.268(0.371,2.396) | 2.564(1.247,5.273) |
| Model 3 | 1 | 2.140(1.200,3.815) | 2.842(1.396,5.783) |
| Model 4 | 1 | 2.023(1.135,3.605) | 2.229(1.071,4.641) |
| Model 5 | 1 | 1.883(1.047,3.387) | 2.364(1.139,4.907) |
| Model 6 | 1 | 1.881(1.022,3.464) | 2.692(1.277,5.674) |
| Model 7 | 1 | 1.612(0.876,2.966) | 2.481(1.189,5.176) |
| Model 8 | 1 | 2.194(1.216,3.959) | 2.740(1.346,5.580) |

a Group 1: both GGT and ferritin were not in the fourth quartile; Group 2: only GGT or ferritin was in the fourth quartile; Group 3: both GGT and ferritin were in the fourth quartile.

Model 1, adjusted for age, Model 2, adjusted for BMI; Model 3, adjusted for drinking, Model 4, adjusted for T2D; Model 5, adjusted for hypertension; Model 6, adjusted for hypertriglyceridemia; Model 7, adjusted for Metabolic syndrome; Model 8, adjusted for MDA.

Table S2 The risks of CKD in groups with the highest quartile of serum GGT or/and ferritin compared with group with other quartiles of serum GGT and ferritin levels in women^a^

|  | Group 1 | Group 2 | Group 3 |
| --- | --- | --- | --- |
| Model 0 | 1 | 1.540(0.989,2.396) | 2.247(1.182,4.272) |
| Model 1 | 1 | --- | 1.840(0.921,3.679) |
| Model 2 | 1 | --- | 2.103(1.125,4.316) |
| Model 3 | 1 | --- | 2.166(1.130,4.152) |
| Model 4 | 1 | --- | 1.551(0.769,3.129) |
| Model 5 | 1 | --- | 1.911(0.978,3.732) |
| Model 6 | 1 | --- | 1.954(0.973,3.927) |
| Model 7 | 1 | --- | 2.004(0.991,4.055) |
| Model 8 | 1 | --- | 2.251(1.172,4.323) |

a Group 1: both GGT and ferritin were not in the fourth quartile; Group 2: only GGT or ferritin was in the fourth quartile; Group 3: both GGT and ferritin were in the fourth quartile.

Model 1, adjusted for age, Model 2, adjusted for BMI; Model 3, adjusted for drinking, Model 4, adjusted for T2D; Model 5, adjusted for hypertension; Model 6, adjusted for hypertriglyceridemia; Model 7, adjusted for Metabolic syndrome; Model 8, adjusted for MDA.
